# Ki-67 Index Provides Long-Term Survival Information for Early-Stage HER2-Low-Positive Breast Cancer: A Single-Institute Retrospective Analysis

**DOI:** 10.1155/2022/4364151

**Published:** 2022-09-13

**Authors:** Wei-Xiang Qi, Lingyan Chen, Lu Cao, Cheng Xu, Gang Cai, Jiayi Chen

**Affiliations:** ^1^Department of Radiation Oncology, Ruijin Hospital, Shanghai Jiaotong University School of Medicine, Shanghai, China; ^2^Clinical Research Unit, Shanghai Ninth People's Hospital, Shanghai Jiao Tong University School of Medicine, Shanghai, China

## Abstract

**Aim:**

It has been reported that more than half of breast cancer (BC) could be identified as HER2-low-positive, which might be a distinct subtype. But the results are controversial. We aim to compare the survival outcomes between HER2-low-positive and HER2-0 BC with Asian women based on HR status or Ki-67 index.

**Methods:**

Between January 2009 and December 2017, HER2-nonamplified BC in our single institute was identified. Patients were classified as HER2-low and HER2-0 cohort. Clinical characteristics were compared between these two groups and survival outcomes were calculated by the Kaplan–Meier method. We also performed subgroup analysis according to Ki-67 index and hormone-receptor (HR) status.

**Results:**

Of the 2,230 included patients, 536 presented with HER2-0, and 1,694 with HER2-low positive. After a median follow-up of 85 months (range: 1–152 months), the 8-year OS, BCSS, and RFS of the overall cohort were 91%, 95%, and 89%, respectively. In comparison with the HER2-0 cohort, majority of HER2-low-expression BC concurrently presented with HR positive (82.3% vs. 69%, *P* < 0.001). There was no significant survival difference between the two groups in terms of OS, BCSS, and RFS (all *p* > 0.05). We then performed subgroup analysis according to HR status and Ki-67 index (<14% vs. ≥14%). Our results indicated that there was no significant survival difference between HER2-low-positive and HER2-0 tumors regardless of HR status (*p* > 0.05), while OS (*p*=0.026) and BCSS (*p*=0.052) of HER2-0 BC with high Ki-67 index were significantly poorer than that of HER2-low positive with high Ki-67, but not for RFS (*p*=0.17).

**Conclusion:**

Among early stage HER2-nonamplified BC, no significant survival difference could be found between HER2-low positive and HER2-0 cohort regardless of HR status. Survival outcomes of HER2-low positive with high Ki-67 seem to be poorer than that of HER2-0 tumors with high Ki-67 index.

## 1. Introduction

Human epidermal growth factor receptor 2 (HER2) is a member of the epidermal growth factor receptor family with tyrosine kinase activity, and dimerization of the receptor initiates the pathways of cell growth, proliferation, and migration [1]. Overexpression of HER2 is an adverse prognostic factor in a majority of cancer types, such as gastric cancer and breast cancer (BC) [2]. Traditionally, results of HER2 expression for tumors is simply defined as positive or negative status. HER2-positive is defined as either IHC 3+ or IHC 2+/FISH(-) [3] based on prevailing HER2 testing guidelines. During the past decades, the availability of HER2-targeted therapy including trastuzumab [4, 5], lapatinib [6, 7], pertuzumab [8–10], and trastuzumab emtansine [11, 12] have significantly improved the outcomes of HER2-positive BC. However, the majority of BC tumors present a low expression of HER2 (IHC 1+ or 2+, but FISH negative), and the recent report of NASBP B-47 shows that no survival benefit could be obtained from the combination of trastuzumab with chemotherapy among women with HER2-low-positive BC [13]. In recent years, more and more evidence indicates that formerly known HER2-negative BC with low or moderate HER2 expression might be a distinct subgroup of BC [14, 15]. However, the prognostic value of HER2-low expression in BC remains controversial. In Rossi V. et al's study, 1,150 early-stage BC patients who underwent curative surgery were analyzed and the authors found that a HER2 2+, but FISH negative was a prognostic indicator for poor prognosis of operable BC patients [16]. Consistent with this result, Eggemann H. et al demonstrated that the prognosis of HR positive with moderate expression of HER2 was significantly poorer than that of HR positive with HER2-0 [17]. However, in a recently published pooled analysis of 2,310 BC patients with HER2 low-moderate expression, the authors showed that 3-year overall survival of HER2-low positive BC was significantly better than HER2-0 BC (91·6% vs 85·8%, *p* = 0.0016) [15]. As a result, the prognostic role of HER2 low-expression for BC patients remains controversial. In addition, there is no published data to compare survival outcomes of HER2-low-positive vs. HER2-0 BC with Asian women. Additionally, multiple studies had indicated that HR status or Ki-67 index might be two important indicators for outcomes of early-stage BC [18–21], but the prognostic role HR status and Ki-67 index in HER2 nonamplified BC is also unknown. Therefore, we conduct this single-institute analysis to assess the prognostic role of HER2-low-positive in Asian women with early-stage BC, and a planned subgroup analysis according to HR status and Ki-67 index is also performed.

## 2. Materials and Methods

From January 2009 to December 2017, there were 2,230 HER2-nonamplified primary BC patients undergoing curative breast surgery including mastectomy or breast conserving surgery with pathological (*P*) N0-1 identified from the department of radiation oncology, Ruijin Hospital. All included patients were needed to fulfill the following criterial: (1) pathological confirmed invasive breast cancer; (2) negative or 1–3 lymph node metastasis; (3) breast cancer among woman; (4). information about HER2 expression, HR status, and Ki-67 index could be available. This study was approved by the local Ethical Committee of our hospital, and the ethical approved number was [2020 (250)]. Because this is a retrospective study, patients' written consent was waved.

### 2.1. Outcomes Definitions

In the present study, HER2 nonamplified BC was classified as HER2 low positive (IHC 1+ or IHC 2+ and FISH negative) and HER2-0 (IHC-0) [15]. Additionally, hormonal receptor expression of ER, PR, and Ki-67 index were also assessed by using immunohistochemistry. Overall survival (OS) was the primary endpoint of the present study. OS was calculated as the time from breast cancer surgery to death or lost to follow-up. Breast cancer specific survival (BCSS) and recurrence-free survival (RFS) were secondary endpoints.

### 2.2. Statistical Analysis

Statistical analyses were conducted through NCSS 11 Statistical Software (2016). We used the *χ* test to compare the baseline characteristics difference between HER2-low-positive and HER2-0 cohorts. Survival rates for OS, BCSS, and RFS were calculated by the Kaplan–Meier method, and the survival comparison was determined using log-rank test between HER2-low positive and HER2-0 groups. Planned subgroups based on HR expression (negative vs. positive) and Ki-67 index values (<14% vs. ≥14%) were also performed.

## 3. Results

### 3.1. Patient Characteristics

Between January 2009 and December 2017, we identified 2,230 consecutively early-stageHER2-nonamplified BC after radical surgery for analysis. Among them, 1,694 patients presented with HER2-low-positive, and 536 with HER2-0. Supplemental Figure 1 shows the process of patient selection and analysis. The patient characteristics of all cohorts are described in Supplemental Table 1. We then compared the baseline characteristics difference among the two cohorts. More patients in HER2-low-positive cohorts were presented with HR positive, treated with hormonal therapy, and breast conserved surgery, when compared to HER2-0 cohort (*p* > 0.05, Table 1). Additionally, patient characteristics such as age, grading, *T* stage, pathological stage, histopathological types, Ki-67 index, adjuvant chemotherapy, and radiotherapy, were comparable between HER2-0 and HER2-low-positive patients. Table 1 lists more information about characteristics of the two cohorts.

### 3.2. Survival Analysis

**Figure 1:**
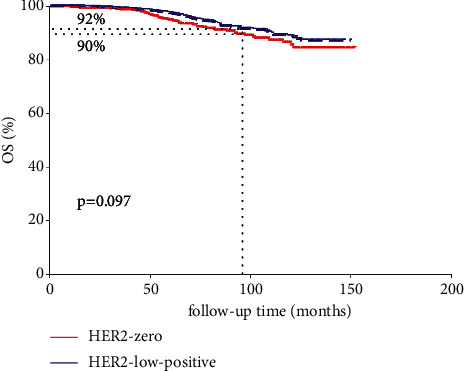
K-M analysis of OS according to HER-2 expression (HER-2 low-expression vs. HER2 0).

**Figure 2:**
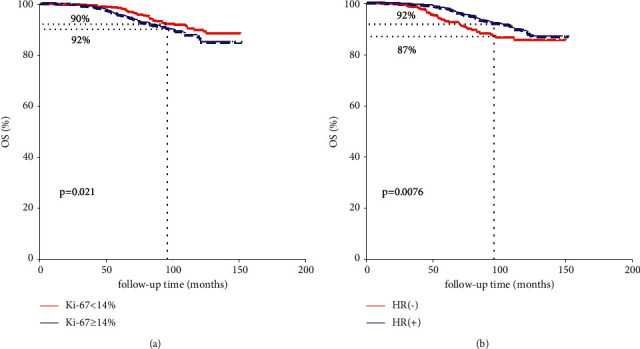
K-M analysis of OS according to Ki-67 index and HR status in the overall population.

The median follow-up for the cohort was 85 months (range: 1–152 months) since the latest follow-up of July 2021, and the 8-year OS, BCSS, and RFS of all cohorts were 91%, 95%, and 89%, respectively. In comparison with the HER2-0 cohort, HER2-low-positive BC was enriched for HR positive tumors (82.3% vs. 69%, *P* < 0.001, Table 2). K-M analysis indicated that OS of HER2-low-positive BC seemed to be better than HER2-0 BC (92% vs. 90%, *p*=0.097, Figure 1), while no significant difference of RFS (*p*=0.33) and BCSS (*p*=0.2) could be found between the two groups. Multiple studies had confirmed that HR status [22] and Ki-67 [23–25] status which could significantly impact the survival outcomes of BC patients. Therefore, we then conducted survival analysis according to HR status (negative vs. positive) and Ki-67 index (<14% vs. ≥14%). Our results showed that OS of HER2-nonamplified BC with low Ki-67 index was statistically better than those HER2-nonamplified BC presented a high Ki-67 index (92% vs. 90%, *p*=0.021, Figure 2(a)). Similarly, OS of HR (+) HER2-nonamplified BC was significantly better than HR (-) HER2-nonamplified BC (92% vs. 87%, *p*=0.0076, Figure 2(b)).

### 3.3. Subgroup Analysis

**Figure 3:**
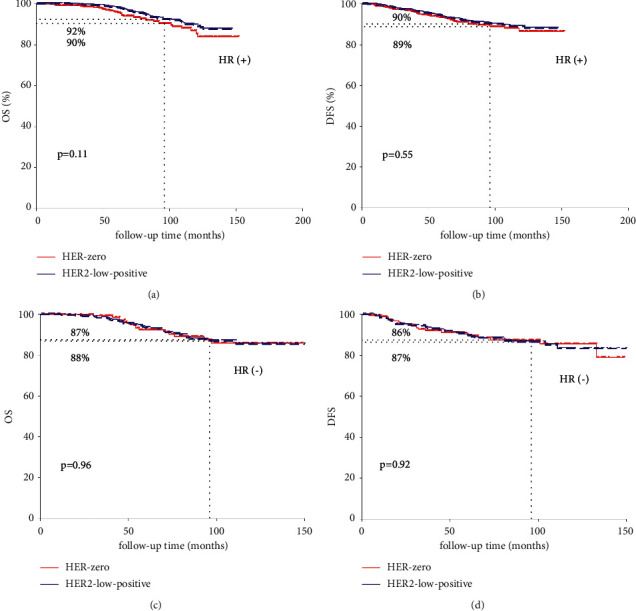
OS and DFS comparison between HER2-low-positive BC and HER2-0 according to HR status.

In HR-positive tumors, no significant survival difference of OS (92% vs. 90%, *p*=0.11Figure 3(a)) and RFS (90% vs. 89%, *p*=0.55, Figure 3(b)) could be observed in HER2-low-positive tumors and HER2-0 tumors. In HR (-) tumors, no significant survival difference of OS (88% vs. 87%, *p*=0.96, Figure 3(c)) and RFS (87% vs. 86%, *p* = 0.92, Figure 3(d)) could be observed in HER2-low-positive BC and HER2-0 BC.

**Figure 4:**
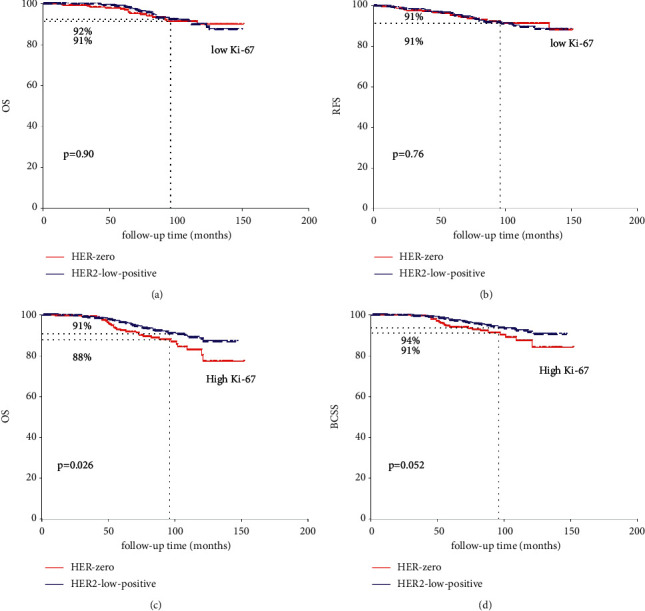
Survival comparison between HER2-low-positive BC and HER2-0 according to Ki-67 index.

Subsequently, we also compared the survival difference between the two cohorts according to the Ki-67 index (<14% vs. ≥14%). In HER2-nonamplified BC with low Ki-67 index, no significant survival difference of OS (92% vs. 91%, *p*=0.90Figure 4(a)) and RFS (91% vs. 91%, *p*=0.76, Figure 4(b)) could be observed in HER2-low-positive BC and HER2-0 BC. In HER2-nonamplified BC with high Ki-67 index, the 8-year OS and BCSS of HER2-low-positive BC was significantly better than HER2-0 BC (91% vs. 88%, *p*=0.026, Figure 4(c); 94% vs. 91%, *p*=0.052Figure 4(d)), while no significant difference of RFS could be observed between the two cohorts (88% vs. 86%, *p*=0.17).

## 4. Discussion

Due to the substantial antitumor activity of novel HER2-targetedantibody-drug conjugates(ADCs) in HER2-low-positive BC, great interest has been increased about the clinical features of this BC subtype [26–28]. Currently, the impact of HER2 low expression on outcome of HER2-nonamplified primary BC remains controversial as HER2-low BC comprising a heterogeneous group. Two previous reports demonstrated that the outcome of BC with HER2 (2+) expression and FISH(−) was poorer than those with HER2-0 BC [16, 17]. However, a recent individual meta-analysis of 2310 patients showed that HER2-low-positive BC had a significant favorable survival than HER2-0 BC(OS: *p*=0.0016), and similar results were detected among HR (−) tumors (OS: *p*=0.016), but not for HR (+) positive BC (*p* = 0.13) [15]. However, significant difference of baseline characteristic including histological grading and Ki-67 index, could be observed between the two cohorts, which could impact patient survival outcomes. In addition, the survival comparison between the two BC cohorts among Asian patients remains unknown. Therefore, we perform the present study to comprehensively compare the characteristics and outcomes between the two cohorts among Asian women patients.

A total of 2,230 HER2-nonamplified breast cancer are identified from our institute. The distributions of most clinical-pathological characteristics between HER2-0 and HER2-low-expression cohorts are comparable, excepting for HR status, and types of surgery. Consistent with previous reports [15, 29], we find that HER2-low-positive BC is enriched for hormone-receptor positive tumors (82.3% vs. 69%, *p* < 0.001). As for survival outcomes of the two cohorts, a tendency of an improved OS could be observed in HER2-low-positive BC in comparison with HER2-0 tumor (*p*=0.097), but no significant difference of BCSS and DFS could be observed between the two groups. Therefore, additional markers are needed to identify high-risk patients in HER2-nonamplified BC.

It has been proposed that certain immunohistochemical (IHC) markers of ER, PgR, HER2, and Ki-67 can provide survival information, which might be equal to those provided by multigenomic analysis including 21 genes [30]. These four markers are readily available routine histopathological parameters. As a result, we further attempt to determine intrinsic subtypes of HER2-nonamplified BC according to HR status and Ki-67 index. Prior to the present study, multiple studies have indicated that Ki-67 index might be significantly related to poor survival for BC patients. Thangarajah F. et al. [31] performed a retrospective analysis and found that disease-free-survival for BC presented with high Ki-67 index was significantly poorer than those with low Ki-67 index (*p*=0.002). Then, Petrelli F. et al. [32] conducted a large systematic review analysis of 64,196 patients and confirmed that Ki-67 index was an independent prognostic indicator for OS, but the optimal cut-off value of Ki-67 index remains undetermined. In a more recent study, the authors found that Ki-67 index was significantly associated with aggressive characteristics [33]. However, the impact of Ki-67 index on outcomes of early-stageHER2-nonamplified BC remains unknown. In our study, we find that HER2-low expression was a prognostic indictor for better OS and BCSS among BC with high Ki-67 index cohort, but not for RFS in comparison with HER2-0. Additionally, among BC with low Ki-67 index, survival difference was comparable between the two cohorts. Therefore, for early-stageHER2-nonamplified BC patients, Ki-67 index could provide additional long-term survival information.

Hormone receptor status might be another factor impact on the survival outcome of this patient population. In consistent with previous study [22], our results show that the OS of HR (+) HER2-nonamplified BC is significantly better than HR (−) HER2-nonamplified BC (92% vs. 87%, *p* = 0.0076). As a result, we perform subgroup analysis HR status. In contrast to previous report by Denkert C. et al [15], our study finds that there is no significant survival difference which could be found between the two cohorts in spite of HR expression. One possible explanation for this finding is that HER2-nonamplified BC is a heterogeneous disease, adding individual genomic data to HER2-low expression cohort might provide additional information to identify patients at high risk, which has been confirmed in Mutai R. et al's study [29]. The authors found that the survival of HER2-low expression among BC with RS > 25 was favorable than HER2-0, while for BC with low genomic risk, there is no association between long-term prognosis and HER2 expression. Further studies to investigating the genetic susceptibility impact on the HER2 expression in breast cancer remains needed.

Our study has four major limitations needed to be concerned. Firstly, selection bias could not be avoided due to the characteristic of the retrospective study, although the baseline characteristic between HER2-0 and HER2-low-positive is comparable. Secondly, genomic information could not be available for the included BC cohort, multiple researches indicated that the impact of survival on HER2-low expression among HER2-nonamplified BC varies across the genomic risk [29, 34]. Thirdly, epidemiological studies show that breast cancer is caused by chronic exposure to natural asbestos and asbestiform fibres, the impact of these factors on occurrence of HER2 low breast cancer remains unknown. Finally, treatment data about the adherence and duration of endocrine therapy and chemotherapy are not documented, which might impact the survival of BC patients.

## 5. Conclusion

Among early-stageHER2-nonamplified BC, a tendency of improved OS could be observed among HER2-low positive in comparison with the HER2-0 cohort, while no significant survival difference could be found between the two cohorts regardless of HR expression. In HER2-nonamplified BC with high Ki-67 index, the OS of HER2-low-expression BC is significantly better than HER2-0 BC. The present study confirms that the value of Ki-67 index could provide additional survival information among HER2-nonamplified BC patients.

## Figures and Tables

**Table 1:** Baseline characteristics of HER2-nonamplified patients.

	HER2-0 patients (*n* = 536)	HER2-low-positive (*n* = 1694)	*p* value
Age, years			
Median, (range)	53, (22–88)	55, (24–92)	*P* = 0.17
<30 year	5 (0.1%)	10 (0.6%)	*P* = 0.06
30-<40 years	60 (11.2%)	137 (8.0%)	
40-<50 years	133 (24.8%)	425 (25.1%)	
50-<60 years	149 (27.8%)	495 (29.2%)	
60–70 years	113 (21.1%)	413 (24.4%)	
≥70 years	76 (14.2%)	214 (12.6%)	

Hormone receptors status			*p* < 0.0001
Negative	166 (31.0%)	299 (17.7%)	
Positive	370 (69.0%)	1395 (82.3%)	

Grading			*P* = 0.10
Grade I	40 (7.5%)	81	
Grade II	177 (33.0%)	734	
Grade III	145 (27.1%)	432	
Unknown	174 (32.4%)	447	

pT stage			*P* = 0.065
pT1mi	1 (0.2%)	9 (0.5%)	
pT1a	28 (5.2%)	57 (3.4%)	
pT1b	86 (16.0%)	278 (16.4%)	
pT1c	207 (38.6%)	733 (43.2%)	
pT2	179 (33.4%)	518 (30.6%)	
pT3	9 (1.7%)	35 (2.1%)	
pTx	26 (4.3%)	64 (3.8%)	

pN stage			*P* = 0.05
Negative LN	407 (75.9%)	1213 (71.6%)	
One positive LN	66 (12.3%)	274 (16.2%)	
Two positive LN	40 (7.5%)	131 (7.7%)	
Three positive LN	23 (4.3%)	76 (4.5%)	

Histologic types			*P* = 0.073
No special type	377 (70.3%)	1306 (77.1%)	
Invasive lobular	12 (2.2%)	41 (2.4%)	
Mucinous	29 (5.4%)	60 (3.5%)	
Other	118 (22.0%)	287 (16.9%)	

Ki-67			*P* = 0.11
≤14.0%	257 (47.9%)	778 (45.9%)	
>14%	279 (52.1%)	916 (54.1%)	

Types of surgery			*P* = 0.026
Mastectomy	344 (64.2%)	1035 (61.1%)	
BCS	192 (35.8%)	659 (38.9%)	

Adjuvant chemotherapy			*P* = 0.31
Yes	290 (54.1%)	872 (51.5%)	
No	246 (45.9%)	822 (48.5%)	

Adjuvant hormonal therapy			*P* < 0.0001
Yes	342 (63.8%)	1340 (79.1%)	
No	194 (36.2%)	354 (20.9%)	

Adjuvant radiotherapy			*P* = 0.12
Yes	287 (53.5%)	840 (49.6%)	
No	249 (46.5%)	854 (50.4%)	

Stage			*P* = 0.63
IA	257 (47.9%)	828 (48.9%)	
IB	2 (0.4%)	7 (0.4%)	
IIA	185 (34.5%)	551 (32.5%)	
IIB	65 (12.1%)	234 (13.8%)	
IIIA	1 (0.2%)	8 (0.5%)	
Unknown	26 (4.9%)	64 (3.8%)	

Abbreviations: LN, lymph node.

**Table 2:** Long-term overall survival of HER2-nonamplified primary breast cancer.

Cohorts	Subgroup	8-yearoverall survival (%)	*p* value
HER2 nonamplified BC	HER2 0	92	0.097
	HER2-low positive	90	

HER2 nonamplified BC	Low Ki-67 index	92	0.021
	High Ki-67 index	90	

HER2 nonamplified BC	HR positive	92	0.0076
	HR negative	87	

HER2 nonamplified BC with HR positive	HER2 0	92	0.11
	HER2-low positive	90	

HER2 nonamplified BC with HR negative	HER2 0	88	0.96
	HER2-low positive	87	

HER2 nonamplified BC with low Ki-67 index	HER2 0	92	0.90
	HER2-low positive	91	

HER2 nonamplified BC with high Ki-67 index	HER2 0	91	0.026
	HER2-low positive	88	

## Data Availability

The datasets used to support the findings of our study could be made available from the corresponding author on reasonable request.

## Supplementary Materials

Supplementary Materials Supplemental table 1: baseline characteristics of included patients.
