# Supplementary material for: Ki-67 Index Provides Long-Term Survival Information for Early-Stage HER2-Low-Positive Breast Cancer: A Single-Institute Retrospective Analysis
Source: J Oncol. 2022 Sep 13;2022:4364151. doi: 10.1155/2022/4364151 (PMC9489376; doi:10.1155/2022/4364151)
Supplement: Supplementary Materials — Supplemental table 1: baseline characteristics of included patients. [file 4364151.f1.zip › supplemental table 1.docx]

**Supplemental table 1: baseline characteristics of included patients**

| **Variable** | N (%) |
| --- | --- |
| **Median age, range, (year)** | 54 (22-92) year |
| **Hormone receptor status** |  |
| **Negative** | 465 (20.9%) |
| **Positive** | 1765 (79.1%) |
| **Stage** |  |
| **IA** | 1085 (48.7%) |
| **IB** | 9 (0.4%) |
| **IIA** | 736 (33.0%) |
| **IIB** | 299 (13.4%) |
| **IIIA** | 9 (0.4%) |
| **Unknown** | 90 (3.0%) |
| **Type of surgery** |  |
| **Mastectomy** | 1379 (61.8%) |
| **BCS** | 851 (38.2%) |
| **Ki-67** |  |
| **≤14.0%** | 1035 (46.4%) |
| **>14%** | 1195 (53.6%) |
| **Adjuvant chemotherapy** |  |
| **Yes** | 1162 (52.1%) |
| **No** | 1068 (47.9%) |
| **Adjuvant hormonal therapy** |  |
| **Yes** | 1682(75.4%) |
| **No** | 548(24.6%) |
| **Adjuvant radiotherapy** |  |
| **Yes** | 1127 (50.5%) |
| **No** | 1103(49.5%) |
