# Supplementary figures and images for: Ki-67 Index Provides Long-Term Survival Information for Early-Stage HER2-Low-Positive Breast Cancer: A Single-Institute Retrospective Analysis
Source: J Oncol. 2022 Sep 13;2022:4364151. doi: 10.1155/2022/4364151 (PMC9489376; doi:10.1155/2022/4364151)

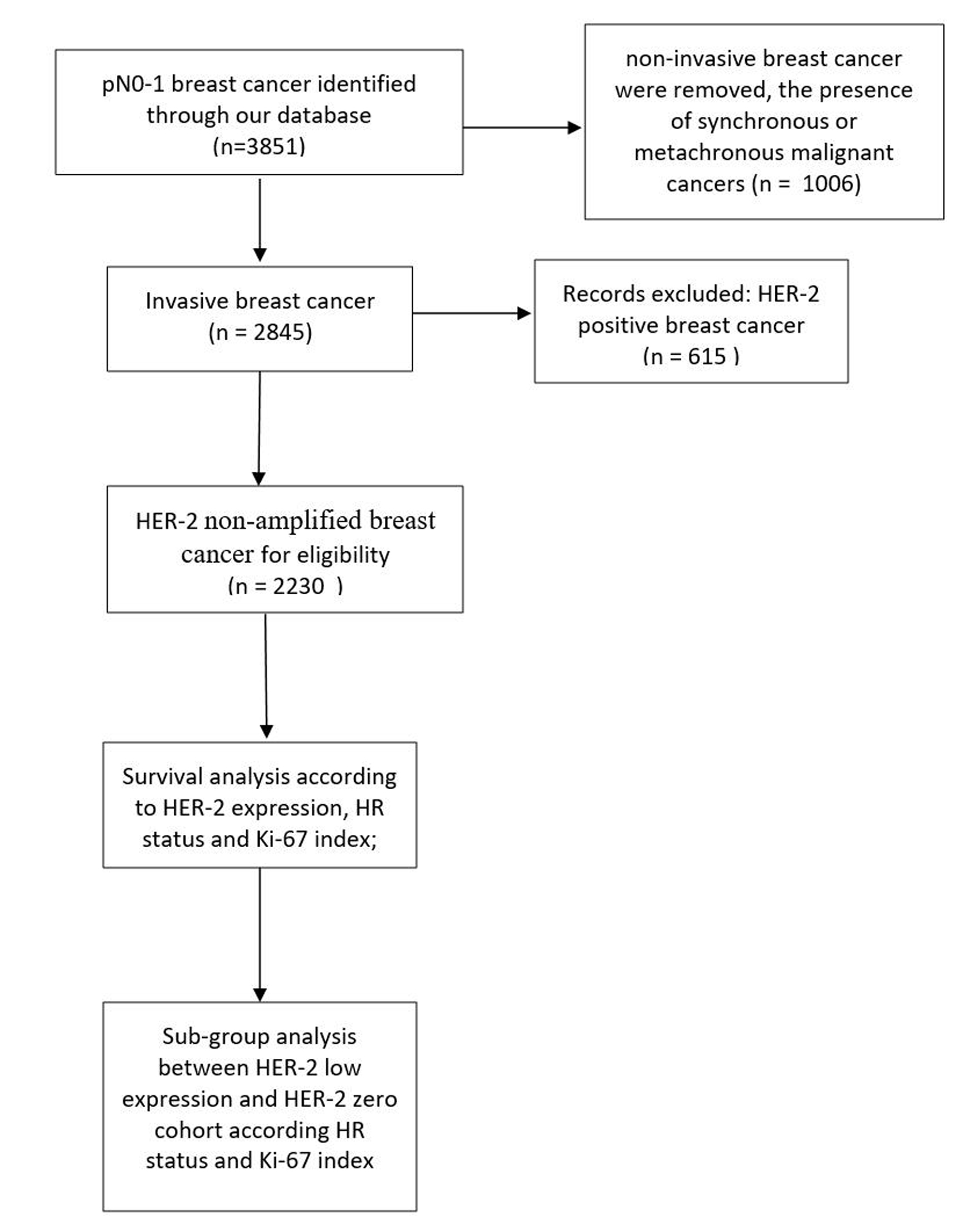

Supplement: Supplementary Materials — Supplemental table 1: baseline characteristics of included patients. [file 4364151.f1.zip › supplemental figure 1.jpg]
